# Selected microRNAs Increase Synaptic Resilience to the Damaging Binding of the Alzheimer’s Disease Amyloid Beta Oligomers

**DOI:** 10.1007/s12035-020-01868-8

**Published:** 2020-01-29

**Authors:** Olga Zolochevska, Giulio Taglialatela

**Affiliations:** Mitchell Center for Neurodegenerative Diseases, Department of Neurology, University of Texas Medical Branch at Galveston, Galveston, TX USA

**Keywords:** Alzheimer’s disease, Non-demented with Alzheimer’s neuropathology, microRNA, Synaptic resilience

## Abstract

Alzheimer’s disease (AD) is marked by synaptic loss (at early stages) and neuronal death (at late stages). Amyloid beta (Aβ) and tau oligomers can target and disrupt synapses thus driving cognitive decay. Non-demented individuals with Alzheimer’s neuropathology (NDAN) are capable of withstanding Aβ and tau toxicity, thus remaining cognitively intact despite presence of AD neuropathology. Understanding the involved mechanism(s) would lead to development of novel effective therapeutic strategies aimed at promoting synaptic resilience to amyloid toxicity. NDAN have a unique hippocampal post-synaptic proteome when compared with AD and control individuals. Potential upstream modulators of such unique proteomic profile are miRNA-485, miRNA-4723 and miRNA-149, which we found differentially expressed in AD and NDAN vs. control. We thus hypothesized that these miRNAs play an important role in promoting either synaptic resistance or sensitization to Aβ oligomer binding. Using an in vivo mouse model, we found that administration of these miRNAs affected key synaptic genes and significantly decreased Aβ binding to the synapses. Our findings suggest that miRNA regulation and homeostasis are crucial for Aβ interaction with synaptic terminals and support that a unique miRNA regulation could be driving synaptic resistance to Aβ toxicity in NDAN, thus contributing to their preserved cognitive abilities.

**Electronic supplementary material:**

The online version of this article (10.1007/s12035-020-01868-8) contains supplementary material, which is available to authorized users.

## Introduction

Alzheimer’s disease (AD), the most common form of dementia and the 6th leading cause of death in the USA, cannot be prevented, cured, or slowed down [1]. AD is a multifactorial disease that is characterized by cognitive decline and unique pathology, senile plaques primarily formed by amyloid beta (Aβ), and neurofibrillary tangles (NFT) of hyper-phosphorylated tau protein that is distinctive for AD-affected brain [2]. However, these pathological changes begin long before memory loss [3] with synapse loss being the most robust correlate of cognitive decline in patients with AD [4, 5]. Synapse loss is believed to occur at early stages of the disease before manifestation of the symptoms ([3, 6] and reviewed by [4, 7]), whereas cell death occurs at later stages [3].

Synaptic dysfunction can occur due to the presence of oligomeric forms of Aβ and tau. During Aβ oligomer interaction with the synapses, as reviewed by Sengupta et al., they can exert their toxic function via multiple mechanisms [8]. For instance, Aβ oligomer association with the post-synaptic densities (PSDs) results in disturbed Ca^2+^ signaling in dendritic spines, which can affect multiple downstream pathways [9]. Moreover, Aβ toxicity can be dependent on size, aggregation state, and diffusion of Aβ oligomers (reviewed by [8]). It was previously demonstrated that, contrary to Aβ oligomers, fibrillar Aβ is incapable of association with the PSDs of primary hippocampal neurons [9]. In fact, there is no correlation between presence of mature Aβ senile plaques with the cell loss or cognitive decline [10–14].

Currently, there is no cure for AD, and despite multiple clinical trials, an effective disease-modifying therapeutic approach is yet to be discovered. Alternative approaches and therapeutic targets are urgently needed to develop successful therapies against AD. A different, possibly more efficient approach in search of novel therapies can be taken stemming from the observation that some individuals, here referred to as NDAN, non-demented with Alzheimer’s neuropathology, are able to retain cognitive function despite the presence of AD-like pathology (reviewed by [15]). Specifically, NDAN individuals show little or no synapse loss [16], along with preserved neurogenesis potentially regulated by microRNAs [17], as well as PSDs that present with a unique proteomic signature [18] and are resistant to Aβ oligomer binding [19], thus protecting synapses from Aβ oligomer–driven dysfunction and likely contributing to maintenance of cognitive ability despite the presence of AD neuropathology. These observations suggest that NDAN individuals are resilient to the cognitive decline that normally ensues as a consequence of accumulation of AD-like neuropathology. It follows that unveiling the yet unclear mechanisms responsible for preservation of cognitive function in NDAN is important as it would support a new treatment strategy centered on inducing cognitive resistance in anyone affected by AD pathology.

Stemming from our previous report describing the presence of a unique post-synaptic proteome in NDAN individuals [18], we aimed to determine the upstream regulators that could be responsible for differential protein levels in NDAN individuals when compared with control and AD. We found three microRNAs (miRs) to be expressed at different levels in control, AD, and NDAN, which are potentially involved in regulation of protein levels at the PSDs. Therefore, in this study, we tested the hypothesis that a global action of miRs allows NDAN synapses to acquire resistance to Aβ oligomer binding. We demonstrate here that these specific miRs decrease Aβ oligomer association with the synapses, possibly by modifying the hippocampal transcriptome.

## Methods

### Case Subjects

Frozen mid-hippocampus tissue was obtained from the Oregon Brain Bank at Oregon Health and Science University (OHSU) in Portland, OR. Donor subjects were enrolled and clinically evaluated in studies at the NIH-sponsored Layton Aging and AD Center (ADC) at OHSU. Subjects were participants in brain aging studies at the ADC and received annual neurological and neuropsychological evaluations, with a clinical dementia rating (CDR) assigned by an experienced clinician. Controls and NDAN had normal cognitive and functional examinations with CDR < 1. The AD subjects were diagnosed by a clinical team consensus conference, met the National Institute for Neurological and Communicative Disorders and Stroke-Alzheimer’s Disease and Related Disorder Association diagnostic criteria for clinical AD, and had a CDR of greater than 1.0 and neuropathologic confirmation at autopsy (after informed consent). Tissue use conformed to institutional review board–approved protocols. Neuropathologic assessment conformed to National Institute on Aging-Reagan consensus criteria. All brain tissue was examined by a neuropathologist for neurodegenerative pathology including neurofibrillary tangles and neuritic plaques. Using standardized CERAD criteria [20], cases were assigned an amyloid score based on the deposition of amyloid plaques in the brain (0 = no plaques, 1 = sparse plaques, 2 = moderate plaques, and 3 = dense plaques) and a Braak stage (0–6; with 6 being the most severe) indicative of the level and location of hyperphosphorylated tau tangles [21]. In addition to the pathological information detailed above, demographical data were received along with the frozen tissue.

### RNA Isolation and Real-Time PCR

RNA was isolated using Trizol Reagent (Life Technologies, Carlsbad, CA) according to the manufacturer’s protocol. Tissue was placed in Trizol and homogenized using the Polytron homogenizer (ThermoFisher Scientific, Waltham, MA). Chloroform was then added, and the samples were spun down at 12,000 rpm for 15 min at 4 °C. The aqueous phase was transferred to a new tube containing isopropanol. The samples were centrifuged at 12,000 rpm for 10 min at 4 °C. Pellet was washed with ice-cold 80% ethanol and air-dried. The samples were resuspended in 40 μl nuclease-free water. The RNA concentration was measured using NanoDrop 2000c (ThermoFisher Scientific, Waltham, MA).

#### miR qPCR

Reverse transcription was performed using miScript II RT Kit (Qiagen, Hilden, Germany) according to the manufacturer’s protocol. Briefly, 0.5 μg RNA was reverse-transcribed in 20 μl reaction volume containing 1x HiSpec buffer, 1x miScript Nucleics Mix, and miScript Reverse Transcriptase. The mix was incubated at 37 °C for 1 h, then at 95 °C for 5 min, and placed on ice. The reverse-transcribed miR mix was diluted with nuclease-free water to a final concentration of 3 ng/μl. Real-time PCR was performed to quantitate miRs in control, AD, and NDAN. miScript SYBR Green PCR Kit (Qiagen, Hilden, Germany) was used according to the manufacturer’s protocol. Briefly, the reaction was performed in 25 μl final volume in each well containing 3 ng reverse-transcribed miR, 1x SYBR Green, and reverse and forward primers (Qiagen, Hilden, Germany). The reaction was performed in Mastercycler epgradient S (Eppendorf, Hamburg, Germany). The samples were incubated at 95 °C for 15 min to activate the polymerase followed by 40 cycles of amplification: 94 °C for 15 s, 55 °C for 30 s, and 70 °C for 30 s. Standard melting curve was performed at the end. All samples were run in duplicate and levels of miRs were normalized to U6 snRNA. The relative fold change in expression of target miRs was determined using the comparative cycle threshold method (2^-ΔΔCt^), and the obtained values were then log2 transformed.

#### mRNA qPCR

cDNA was made using amfiRivert Platinum cDNA Synthesis Master Mix (GenDEPOT, Katy, TX) according to the manufacturer’s protocol. Briefly, 0.5 μg RNA was first incubated at 70 °C for 5 min and then chilled on ice. The cDNA reaction mix was prepared with the buffer and enzyme mixes provided in the kit. cDNA was made using the following conditions: 25 °C for 5 min, followed by incubation at 42 °C for 60 min and finally 15 min at 70 °C.

The primer sequences were obtained from the PrimerBank (pga.mgh.harvard.edu/primerbank, Harvard, Cambridge, MA) to measure expression of genes of interest. Quantitative real-time PCR (qRT-PCR) was performed to measure mRNA levels. Each well of 96-well plate for qRT-PCR contained 20 ng RNA, 1 mM oligo, and 1x KAPA SYBR FAST Universal qPCR Kit (KAPA Biosystems, St. Louis, MO). All samples were run in duplicate; standard melting curve was performed at the end. Measured mRNA values were normalized to the expression level of actin. The relative fold change in expression of mRNAs was determined using the comparative cycle threshold method (2^-ΔΔCt^), and the obtained values were then log2 transformed.

### Animals

Eleven- to 13-week-old wild-type male and female C57B6 mice were purchased from the Jackson Laboratory (Bar Harbor, ME). Health care for all animals was provided by the animal care specialists under a supervision of the facility manager. The care and maintenance were provided for the animal colony on a daily basis to ensure the safe and healthy environment. Each animal was used under an animal protocol approved by the Institutional Animal Care and Use Committee of the University of Texas Medical Branch, ensuring that the animals received the minimal amount of pain/discomfort. All animals were housed under USDA standards (12:12-h light/dark cycle, food and water ad libitum) at the University of Texas Medical Branch vivarium.

### ICV Injections

Male and female mice were injected intracerebroventricularly (ICV) with miRs (scrambled, 149, 485, and 4723) (ThermoFisher Scientific, Waltham, MA) dissolved in artificial cerebrospinal fluid. Seven animals per group were used.

ICV injection is a technique routinely used by our laboratory [22]. Briefly, mice were anesthetized with isoflurane. The ICV injections were performed according to the freehand injection method described by Clark et al. [23]. Twenty-nine-gauge needle was held with hemostatic forceps to leave 4 mm of the needle tip exposed. The needle was connected to a 25-μl Hamilton syringe via 0.38-mm polyethylene tubing. The injection volume was set at 2 μl to deliver 1 nmole of miR; infusion rate was set at 1 μl/min using electronic programmable microinfuser (Harvard Apparatus, Cambridge, MA). After the injection, the needle was left in place for 1 min. The mouse was allowed to recover while lying on a heated pad under warm light. Twenty-four hours post-injection, mice were euthanized by using deep anesthesia followed by cervical dislocation. Mouse brain was quickly collected and stored at − 80 °C for further analysis.

### Isolation of Synaptosomes

Synaptosomes were isolated using Syn-PER Reagent (ThermoFisher Scientific, Waltham, MA) according to the manufacturer’s protocol. Briefly, approximately 30 mg of tissue was homogenized using Dounce glass homogenizer in the presence of Halt Protease Inhibitor Cocktail (ThermoFisher Scientific, Waltham, MA) and Phosphatase Inhibitor Cocktail (MilliporeSigma, Burlington, MA). The homogenate was spun down at 1200×*g* for 10 min at 4 °C. The supernatant was centrifuged at 15,000×*g* for 20 min at 4 °C to obtain the pellet of synaptosomes. The pellet was then resuspended in HBK (HEPES-buffered Krebs-like) buffer as described before [24]. The concentration of synaptosomes was determined using flow cytometry. The samples were stored at − 80 °C until use. Synaptosome preparations are routinely analyzed by Western blot and electron microscopy to ensure the quality of the preparation, as we have previously reported [24].

### Aβ Oligomer Preparation

Aβ oligomer preparation is a technique, used routinely by our laboratory [19]. Briefly, lyophilized Aβ1–42 aliquots (Department of Biophysics and Biochemistry, Yale University, New Haven, CT) were dissolved in 200 μl of 1,1,1,3,3,3-hexafluoro-2-propanol and then added to 700 μl of distilled deionized H_2_O in microcentrifuge tubes. Loosely capped tubes were stirred on a magnetic stirrer in a fume hood for 48 h and then aliquoted and stored at − 80 °C. In order to prepare labeled Aβ oligomers, a small aliquot of HiLyte™ Fluor 647-labeled Aβ1–42 (AnaSpec, Fremont, CA) was added to the HFP-Aβ mix described above. Western and dot blot analysis using A-11 antibodies (Aβ oligomer specific) are used to determine the quality of oligomerization (as previously described by [25]).

### Ex Vivo Aβ Oligomer Binding and Flow Cytometry

To determine the amount of Aβ oligomers associated with the synaptosomes (synaptosome isolation is described above), two million synaptosomes were incubated with 2.5 μM HiLyteTM Fluor 647-labeled Aβ oligomers for 1 h at room temperature in dark. The samples were washed three times in HBK buffer to remove all unbound Aβ oligomers and resuspended in PBS without Ca^2+^/Mg^2+^. The samples were analyzed using Guava easyCyte flow cytometer (Luminex Corporation, Austin, TX). Standard size polystyrene particles (Spherotech, Inc., Lake Forest, IL) were used to set up size 1– 5 μm gate for synaptosome analyses.

### RNA-Seq

#### Library Construction and Sequencing

Quality of the purified RNA was assessed by visualization of 18S and 28S RNA bands using an Agilent BioAnalyzer 2100 (Agilent Technologies, CA); the electropherograms were used to calculate the 28S/18S ratio, and the RNA integrity number. Poly-A+ RNA was enriched from total RNA (~ 1 μg) using oligo dT-attached magnetic beads. Bound RNA was fragmented by incubation at 94 °C for 8 min in 19.5 μl of fragmentation buffer (Illumina, San Diego, CA). First- and second-strand synthesis, adapter ligation, and amplification of the library were performed using the Illumina TruSeq RNA Sample Preparation Kit as recommended by the manufacturer (Illumina, San Diego, CA). “Index tags” incorporated into the adapters were used to track samples. Library quality was evaluated using an Agilent DNA-1000 chip on an Agilent 2100 Bioanalyzer. Quantification of library DNA templates was performed using qPCR and a known-size reference standard. Sequencing was performed by the UTMB Next Generation Sequencing Core Facility on an Illumina NextSeq 550 with 3 samples per group. Sequencing conditions were paired-end 75 base in the high-output mode.

### RNA-Seq Analysis

The alignment of NGS sequence reads to the mouse mm10 reference genome was performed using the Spliced Transcript Alignment to a Reference (STAR) program, version 2.5.4b [26], using the ENCODE standard options as recommended by the developer. The UCSC version of the mouse reference sequence and annotation files were downloaded from the iGenomes website maintained by Illumina (https://support.illumina.com/sequencing/sequencing_software/igenome.html). The “-quantMode GeneCounts” STAR option was used to count the number or reads mapping to each gene. Differential gene expression was analyzed with the program DESeq2, version 1.18.1, running in R version 3.4.3 [27]. A table of read counts per gene per sample was provided to DESeq2 and differential expression between conditions was tested using the standard analysis vignette provided by the authors.

### Statistical Analysis

PCR data were log2 transformed before statistical analysis. The results were expressed as mean ± standard error unless otherwise noted. Data analysis was completed using GraphPad Prism version 7.05 for Windows (GraphPad Software, La Jolla, California, www.graphpad.com). One-way or two-way ANOVA was performed, followed by either Dunnett’s or Sidak’s multiple comparisons test (specified in the results). A *p* value of less than 0.05 was considered statistically significant.

## Results

### Upstream Regulators of Post-synaptic Proteome Changes in NDAN

We have recently reported the unique protein signature present at the post-synaptic densities of NDAN when compared with AD and age-matched control individuals [18]. As part of these studies, we utilized a bioinformatics approach (Ingenuity Pathway Analysis (IPA)) to determine the upstream drivers of the observed changes. The Upstream Regulator tool of the IPA can identify key upstream players which could elicit the changes observed at the protein level. Following this approach, three miRs were identified as major drivers of the proteome changes observed at the PSDs of NDAN subjects: miR-4723, miR-149, and miR-485. Notably, a literature search revealed that these miRs are all involved in regulation of synaptic genes.

**Fig. 1:**
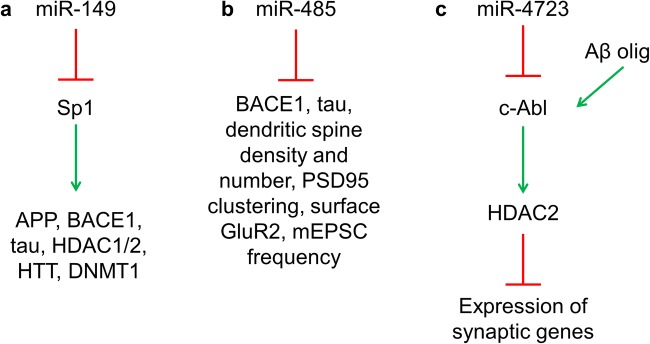
Functions of miR-149, miR-485, and miR-4723. miR-149 (**a**), miR-485 (**b**), and miR-4723 (**c**) are involved in regulation of synaptic genes. Sp1 specificity protein 1, APP amyloid precursor protein, BACE1 beta-secretase 1, HDAC1/2 histone deacetylase 1/2, HTT huntingtin, DNMT1 DNA methyltransferase, PSD95 post-synaptic density protein 95, GluR2 glutamate receptor 2, mEPSC miniature excitatory post-synaptic currents, c-Abl Abelson tyrosine-protein kinase 1. References are provided in text

miR-149 (Fig. 1a) regulates specificity protein 1 (Sp1) [28], APP (amyloid precursor protein), BACE1 (beta-secretase 1), tau, HDAC1/2 (histone deacetylase 1 and 2), huntingtin, and DNMT1 (DNA methyltransferase) [29]. miR-485 (Fig. 1b) regulates the expression of BACE1, tau, dendritic spine density and number, PSD95 (post-synaptic density protein 95) clustering, surface GluR2 (glutamate receptor 2), and the miniature excitatory post-synaptic current frequency [30–32]. miR-4723 (Fig. 1c) downregulates c-Abl (Abelson tyrosine-protein kinase 1) [33], which can also be upregulated directly by Aβ oligomers [34]; c-Abl can regulate the expression of synaptic genes via HDAC2 [35].

**Fig. 2:**
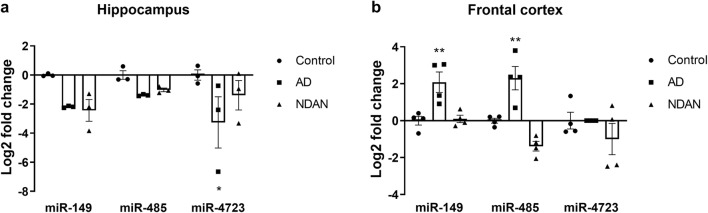
miR levels in the hippocampus of control, AD, and NDAN. IPA-predicted miR-149, miR-485, and miR-4723 are differentially regulated in post-mortem hippocampi (**a**) and frontal cortices (**b**) of AD and NDAN when compared with control, which is set at zero. miR-4723 was below detection limit in AD frontal cortex. Measured miR values were normalized to the expression level of U6. Values represent the means ± SEM. *n* = 4 frontal cortex, *n* = 3 hippocampus. *p < 0.05 and **p < 0.01 vs. control, two-way ANOVA, followed by Dunnett’s multiple comparisons test

Next, to determine the levels of these three IPA-predicted miRs, we isolated RNA from post-mortem hippocampi and frontal cortices of control, AD and NDAN (case subject data is provided in Table 1). We found that, as predicted by the IPA, the three miRs are indeed differentially regulated in both the hippocampus and frontal cortex of AD and NDAN when compared with control subjects (Fig. 2). Interestingly, in AD hippocampus, miR-4723 was significantly decreased (Fig. 2a), while in the frontal cortex, it was below detection limit when compared with control (Fig. 2b). In the frontal cortex, miR-149 and miR-485 were significantly upregulated in AD when compared with control (Fig. 2b). NDAN, on the other hand, had a non-significant trend towards reduction of the three miRs in the hippocampus and frontal cortex when compared with control (Fig. 2). These results suggest that the three miRs may play a role in the progression of AD and can potentially be one of the mechanisms providing resistance to clinical manifestation of the disease for some individuals.

**Table 1:** Demographic data of the cases used to determine levels of miRs. *PMI* post-mortem interval, *FC* frontal cortex, *H* hippocampus

Case number	Diagnosis	Brain region analyzed	Age (years)	Sex	PMI (hours)	Braak stage
767	Control	FC	86	F	8	2
785	Control	FC	83	M	14	1
1957	Control	H	> 89	F	8	4
1965	Control	H	> 89	F	5.5	2
1977	Control	FC	> 89	F	4	4
2229	Control	FC, H	71	F	14.5	2
1969	AD	FC, H	67	F	13	6
2010	AD	FC, H	87	F	6	3
2305	AD	FC, H	85	F	5	6
2318	AD	FC	74	F	2	6
697	NDAN	FC, H	> 89	M	5	5
1016	NDAN	FC	> 89	F	8	6
1179	NDAN	FC, H	89	F	2.5	5
1284	NDAN	FC	> 89	M	72	5
1362	NDAN	H	> 89	F	48	4

### Aβ Oligomer Binding to the Surface of SH-SY5Y

We then tested whether these three miRs had an effect on Aβ oligomer binding to neuronal cells in vitro. We utilized SH-SY5Y cells, a human neuroblastoma cell line, which expresses immature neuronal markers [36]. Cells were transfected with the miRs and 48 h later collected with 10 mM EDTA (to preserve membrane proteins) and then challenged with 2 μM HiLyte^TM^ Fluor 647-labeled Aβ oligomers ex vivo. The cells were also co-transfected with FAM-labeled control siRNA to allow for measurement of Aβ binding only in miR-transfected cells. The Aβ oligomer binding to the cellular surface was assessed by flow cytometry analysis; representative flow cytometry acquisitions are provided in Supp. Fig. 1. As can be seen from Supp. Fig. 2, treatment with miR-485 and miR-4723 resulted in a significantly decreased amount of Aβ oligomers associated with the SH-SY5Y surface, while the transfection with miR-149 had no effect on sensitivity to Aβ oligomers. These results suggest that miR-485 and miR-4723 promote resilience to Aβ oligomer binding in SH-SY5Y.

### Effect of miRs on Aβ Oligomer Binding to Synaptosomes

Administration of these three miRs in vitro to SH-SY5Y human neuroblastoma cells (Supp. Figs. 1 and 2) suggested that these molecules can provide resistance to Aβ oligomers, therefore, we aimed to test their effect in vivo. In order to determine if the in vivo administration of these miRs had an effect on Aβ oligomer binding to the synapses, wild-type C57BL/6 male and female mice received a single ICV injection of the selected miRs. Scrambled miR was injected as a control. At 24 h post-injection, the hippocampi and frontal cortices were collected for analysis, and synaptosomes were isolated. Synaptosomes were challenged ex vivo with 2.5 μm Aβ oligomers as described in the “Methods” section. Flow cytometry was used to assess the extent of the Aβ binding on to the isolated synaptosomes.

**Fig. 3:**
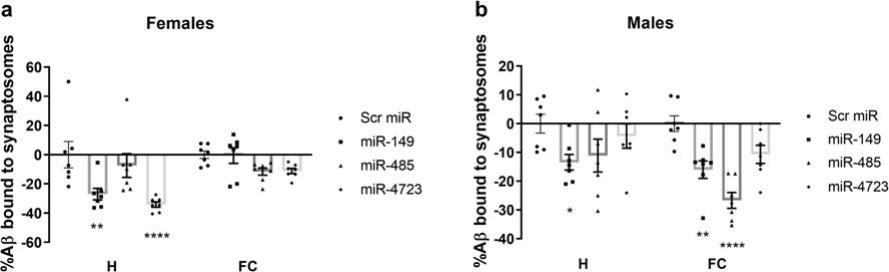
Aβ oligomer binding to synaptosomes in mice after ICV treatment with miR-149, miR-485, and miR-4723. Female (**a**) and male (**b**) mice were injected ICV with the selected miRs; scrambled miR was used as control. Synaptosomes were isolated from hippocampi (H) and frontal cortices (FC) and incubated ex vivo with 2.5 μM tagged Aβ oligomers and analyzed using flow cytometry. Levels of binding to scrambled-injected mice were set at zero. *n* = 7. Values represent the means ± SEM. *p < 0.05, **p < 0.01, and ****p < 0.0001 vs. scrambled miR, two-way ANOVA, followed by Dunnett’s multiple comparisons test.

In order to analyze synaptosomes using flow cytometry, the 2-, 3-, 5-, and 7-μm standard size beads were used to set up the flow gates. Representative acquisitions are provided in Supp. Fig. 3. The synaptosome gate was set up to include ~ 1–5-μm particles, which is the typical size of synaptosomes, as previously described by others [37, 38]. When we analyzed the Aβ binding to the synaptosomes isolated from female hippocampi and frontal cortices (Fig. 3a), we observed that injection of the selected miRs in vivo resulted in significantly decreased binding after treatment with miR-149 and miR-4723 only in the hippocampus. Synaptosomes isolated from the frontal cortex of females, on the other hand, had unaltered Aβ oligomer binding after treatment with miR-149 or miR-4723 when compared with scrambled miR (Fig. 3a). miR-485 had no effect on Aβ binding in neither region analyzed. In contrast to females, males responded to treatments with miR-149 and miR-485 by significantly decreasing the amounts of Aβ bound to the synaptosomes isolated from the frontal cortex (Fig. 3b). On the other hand, miR-4723 had no significant effect on the ability of male synaptosomes to bind Aβ oligomers.

### Hippocampal Transcriptome Changes in Response to miRs

**Fig. 4:**
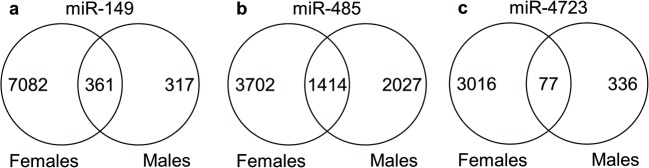
Changes in the hippocampal transcriptome after treatment with miR-149, miR-485, and miR-4723. RNA changes in the hippocampi of miR-treated animals were normalized to mice injected with scrambled miR. The Venn diagram shows an overlap in RNA changes in males vs. females. *n* = 3 mice/group. The Venn diagrams were built using the online Venny tool [69]

Since we observed significant changes in Aβ binding to the synaptosomes isolated from hippocampi of miR-treated mice (Fig. 3), we decided to perform RNA-Seq to determine potential mechanisms providing resistance to Aβ oligomer binding in these mice. RNA-Seq was performed on three samples from each group, and all readings were normalized to the scrambled miR-injected group. For the analysis, the RNA transcripts were selected using the following criteria: log2 fold change ≥ ± 1 and *p* value < 0.05. When we analyzed the hippocampal transcriptome of miR-treated mice, we noticed that each miR engages with its target mRNAs in male and female mice, some of which are shared between two sexes and some are uniquely changed in either males or females alone. Interestingly, a greater number of transcripts was altered in females vs. males after each treatment with the three miRs (Fig. 4). Thus, in response to miR-149 injection 7443 transcripts were altered in females and 678 were changed in males, 361 of which were common to both sexes (Fig. 4a). After the treatment with miR-149 in females, 3606 genes were upregulated and 3837 were downregulated, while in males, 409 were increased and 269 decreased. miR-485 induced changes in 5116 RNAs in females and 3441 genes in males, 1414 of which were common to both groups (Fig. 4b). Injection of miR-485 in females resulted in increased levels of 2591 transcripts and downregulation of 2525 mRNAs, while in males, 1783 were upregulated and 1658 downregulated. Treatment with miR-4723 resulted in 3093 transcripts to be altered in females and 413 in males, 77 of which were common for both sexes (Fig. 4c). miR-4723 upregulated expression of 1273 mRNAs and downregulated 1820 transcripts in females, while in males 250 were increased and 163 were decreased.

**Fig. 5:**
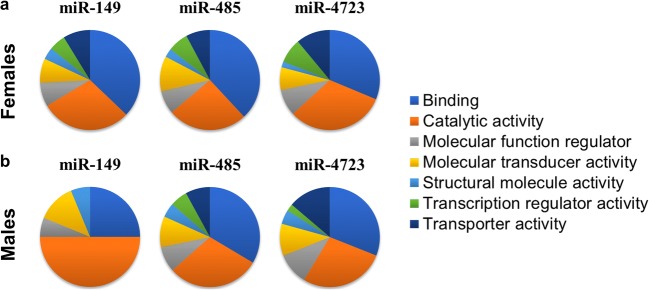
Hippocampal transcriptome analyzed with PANTHER. PANTHER [70, 71] was used to analyze the molecular functions of mRNAs changed after **a** female and **b** male mice were treated ICV with miR-149, miR-485, and miR-4723. Changes in miR-treated animals were normalized to mice injected with scrambled miR

When the hippocampal transcriptome was evaluated using PANTHER, we noticed that overall, the three treatments had some similarities when the RNA changes were analyzed by the molecular function (Fig. 5). The number of mRNAs in each category is provided in Supp. Table 1. According to this analysis, RNAs that changed in response to each miR treatment represent several functions; however, two major categories shared by all transcripts are binding and catalytic activity.

### Expression Levels of Synaptic Genes After miR Treatment

**Fig. 6:**
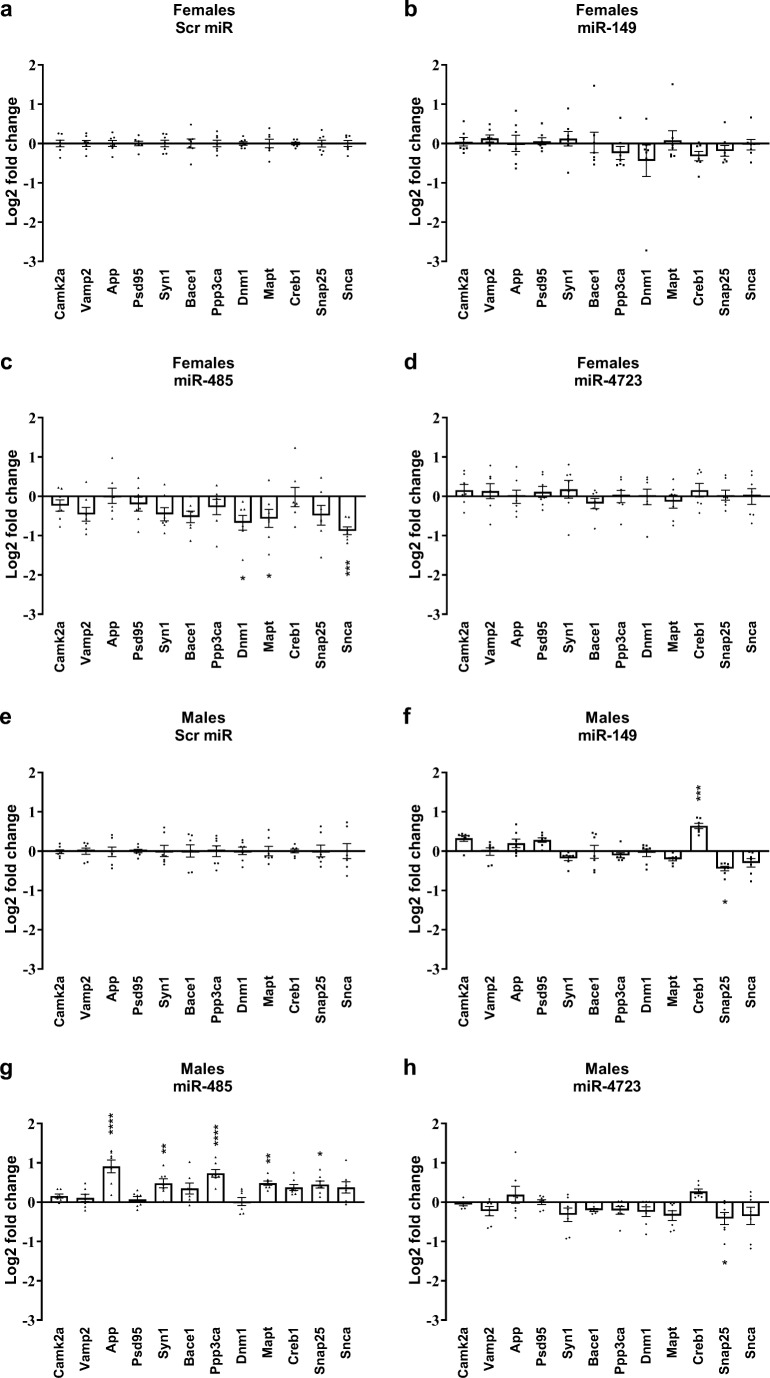
Expression of synaptic genes in hippocampi after treatment with miR-149, miR-485, and miR-4723. Several genes involved in synaptic function were assessed using qRT-PCR in hippocampi obtained from **a**–**d** female or **e**–**h** male mice treated with miRs. Mice treated with scrambled miR were used as a control and set at zero. Measured mRNA levels were normalized to the expression level of actin. Values represent the means ± SEM. *n* = 7. *p < 0.05, **p < 0.01, ***p < 0.001, ****p < 0.0001 vs. scrambled miR, two-way ANOVA, followed by Dunnett’s multiple comparisons test.

To further investigate if the selected miRs have an effect on the expression levels of the genes that are important for synaptic function, we elected to determine an expression profile of twelve such synaptic genes. It is well-documented that Aβ oligomers cause synaptic dysfunction (reviewed by [39]); moreover, Aβ oligomers have multiple docking partners at synaptic terminals [40]. In the present study we have observed decreased Aβ oligomer binding to the synapses after administration of miRs, which then led us to question if these miRs modify genes related to the synaptic function. In order to determine whether this was indeed the case, we measured the levels of several synaptic genes in the hippocampus of mice injected ICV with miR-149, miR-485 and miR-4723 as compared with control mice injected with the scrambled RNA (description of the genes is provided in Supp. Table 2). Twelve genes were selected and quantified using qRT-PCR in hippocampi (Fig. 6). Interestingly, the mRNA levels were completely opposite in males vs. females. Thus, in males, miR-485 upregulated the levels of selected genes, in particular, App, Syn1, Ppp3ca, Mapt, Snap25, and Snca, which were significantly increased compared with control. On the other hand, in females, the same miR-485 (Fig.6c) downregulated Dnm1, Mapt, and Snca. The remaining two miRs (149 and 4723) did not elicit any significant changes in females (Fig. 6b, d). In males, Snap25 was the only gene that was significantly downregulated by miR-4723 (Fig.6h) and miR-149 (Fig.6f), while Creb1 was significantly increased in response to miR-149 (Fig. 6f). These results indicate that the three miRs in our study target different genes, which could potentially translate into different degree of protection against Aβ oligomer binding.

## Discussion

Alterations in miR levels have been associated with AD previously ([41–45] reviewed by [46, 47]) due to the link between multiple families of miRs and hallmark pathological processes in AD, as well as other neurodegenerative disorders ([48] reviewed by [49, 50]). miRs are non-coding 18–22 nucleotide-long single-stranded RNAs that can target multiple messenger RNAs (mRNAs) via Watson-Crick base pairing, leading to their degradation or translational repression. miRs are involved in multiple biological pathways, and their expression is regulated by enzymes which process and stabilize mature miRs, or by epigenetic mechanisms such as DNA methylation or histone modifications [51]. It is hypothesized that in neurodegenerative diseases, miRs can modulate the levels of toxic proteins by modulating the expression of their mRNAs or by regulating mRNA of proteins that regulate the levels of toxic proteins [52].

miRs are extremely potent molecules, regulating thousands of genes and hundreds of networks, known to be involved in multiple stages of AD pathogenesis ([41–45], reviewed by [46, 47, 49, 50]). Here, we focused on three miRs that were selected based on the analysis of the post-synaptic density proteome of NDAN vs. AD and healthy age-matched control individuals [18]. miR-149, miR-485 and, miR-4723 were identified by the IPA as the drivers of differential protein expression at the PSDs of NDAN vs. AD, previously reported by our group [18]. Interestingly, these miRs predicted by IPA are all involved in the regulation of genes involved in synaptic function [29–32, 35].

We show that while all these miRs demonstrated a trend towards a decrease in the hippocampus of AD patients, in the frontal cortex from the same patients miR-149 and miR-485 were significantly increased, whereas miR-4723 was significantly decreased in AD hippocampus. On the other hand, in NDAN these miRs showed a trend towards decrease in both the hippocampus and frontal cortex as compared with control subjects. Hence, the IPA-predicted miRs, involved in regulation of synaptic genes, are differentially expressed in post-mortem human frontal cortices and hippocampi of control, AD and NDAN, thus suggesting that these miRs could potentially be involved in providing synaptic resilience against Aβ oligomers as seen in NDAN individuals [19].

Aβ oligomers are known to disrupt integrity of synapses [22, 53], and multiple miRs have been reported to play key roles in synaptic function and plasticity (i.e. miR-9, −132, −134, −138, −125 and other) (reviewed by [54–57]). MiRs are capable of regulating both functional and structural plasticity at the synapse, thus impacting neural development, physiological function, and possibly disease pathogenesis. Moreover, an interplay between Aβ oligomers and miRs has been described; for instance, Schonrock et al. showed that 47% of all miRs they have tested were rapidly downregulated after treatment with Aβ oligomers [58]. Similar to this published evidence, in our study, we observed a downregulation of endogenous miR-149, miR-485, and miR-4723 after treatment with Aβ oligomers in SH-SY5Y cells (data not shown). It is then tempting to speculate that the balance and fine regulation of miR levels are important factors that can provide resistance or increased sensitivity of synapses to Aβ oligomers. Consistent with this view, we found reduced binding of Aβ oligomers to the cellular surface of cultured human SH-SY5Y neuroblastoma when the cells were treated with miR-485 and miR-4723, although miR-149 was not effective.

Most importantly, we observed a similar resilience to Aβ oligomers when wild-type mice received these miRs ICV. A significant reduction of Aβ oligomer binding to synaptosomes isolated from hippocampi and frontal cortices of miR-treated mice was detected, and such effects appeared to be sex-dependent. miR-485 was more potent at providing protection against Aβ oligomers in males, while miR-4723 treatment resulted in less binding in females. Surprisingly, despite miR-149 effectiveness in the male hippocampus and frontal cortex, in females, it provided protection against Aβ oligomers only in the hippocampus. Furthermore, in females, the hippocampus appeared to be more responsive/sensitive to alterations in miRs, while in males this was true for the frontal cortex. On the other hand, treatment with miR-485 in females and miR-4723 in males did not cause any significant changes in Aβ oligomer synaptic binding when compared with control.

The brain-region differences in gene expression described here are consistent with what has been reported by others (reviewed by [47]). Sex-specific differences in mRNA expression patterns in the brain have been described previously [59–62]; however, the sex-specific sensitivity to Aβ oligomer binding and miR treatments are novel observations. Furthermore, the sex-specific differences described in this manuscript highlight the importance of including (whenever possible) both males and females in the preclinical research, since determining the inherent differences between two sexes could help guide the development of clinical trials.

In order to understand the mechanisms behind the protection against Aβ oligomers provided by these miRs, we performed deep RNA sequencing to determine overall mRNA network changes after treatments with miR-149, miR-485, or miR-4723. Using this approach, we found that each of these miRs modifies a distinct set of mRNAs, and this regulation is sex-specific with relatively small number of shared transcripts. Nevertheless, when each miR treatment was analyzed using PANTHER on the basis of the molecular function, the majority of the mRNAs collectively modified by the miR treatments converged onto binding and catalytic activity functions in addition to a small fraction of RNAs that belong to molecular function regulators and molecular transducers.

It is important to emphasize here that the miR-149, miR-485, and miR-4723 engage multiple mRNAs and potentially have an effect on numerous networks, which altogether provide resistance against Aβ oligomers binding when administered in vivo, as discussed above. This observation suggests that resistance to Aβ oligomers is in tight relationship with the miR homeostasis, supported by our observation of the altered levels of these three miRs in NDAN, individuals whose synapses do not engage in oligomer binding. Thus, Aβ oligomers, miR levels, and synaptic plasticity appear to be intimately interconnected and possibly dependent on each other. While synaptic activity stimulates the production of Aβ (reviewed by [53]), Aβ oligomers can affect miR homeostasis. In turn, while miRs are known to be also regulated by neuronal activity (reviewed by [54]), synaptic plasticity is highly dependent on proper levels of critical miRs.

Furthermore, we have measured levels of a limited set of mRNAs that are either relevant to synaptic function [53, 63–65], are known docking sites of Aβ oligomers [40], or are predicted targets of our miRs (miRWalk database [66, 67]). We found that overall, the miR-149, miR-485, and miR-4723 had opposite effects on selected genes in males vs. females. For example, in males, miR-485 significantly upregulated the synaptic genes measured, including App, Syn1, Ppp3ca, Mapt, Snap25, and Snca, while the same miR in females significantly downregulated Dnm1, Mapt, and Snca. Treatment with miR-4723 in females did not result in any significant changes among the selected genes, whereas in males, miR-4723 downregulated Vamp2, Syn1, Bace1, Dnm1, Mapt, Snap25 and Snca; however, App was increased. miR-149 did not significantly alter the levels of selected genes in either sex, with the exception of Creb1 increase in males. Thus, three selected miRs had sex-specific effects on the twelve synaptic genes that were evaluated in our study. Additionally, more information could be obtained if individual cellular compartments are analyzed since miRs were shown to play distinct roles in the cytoplasm vs. nucleus where they can inhibit or activate their target genes (reviewed by [68]). In fact, it has been previously documented that the effect of individual miR on its target mRNA can be quite small (up to twofold, as seen in our results), but network misregulation in response to miR treatment could reveal effects of greater magnitude (reviewed by [47]). This suggests that analysis of global network responses rather than individual mRNAs can provide more insights into the mechanisms behind a particular functional phenomenon, as shown above for RNA-Seq analysis of female and male hippocampi.

The complexity of miR-driven gene regulation in various cellular compartments in addition to different degrees of up- or downregulation provided by these small potent molecules infers that even modest alterations in miR could profoundly affect overall. However, it remains unclear if dysregulation of miR levels is a cause or a consequence of the disease state. Nonetheless, while more research needs to be done to determine the exact miR targets and study pathways evoked by application of miRs, in this work, we presented strong evidence of reduced Aβ oligomer binding to the synaptic terminals in response to treatment with selected miRs that could be involved in modulation of synaptic resilience to AD neuropathology in NDAN individuals.

## Conclusion

In the present work, we focused on three miRs that were predicted to be upstream drivers of the changes in the post-synaptic proteome that we previously reported in non-demented individuals with AD-like neuropathology who have synapses resilient to the detrimental binding of amyloid oligomers. We found that, although with varying efficiency, all three miRs (miR-149, miR-485, and miR-4723) were capable of increasing synapse resilience to Aβ oligomers when delivered in vivo ICV in adult mice, possibly via modulating the expression of key mRNAs. Interestingly, we found these protective effects to be brain region– and sex-dependent. While to the best of our knowledge this is the first evidence of synaptic resilience to oligomers regulated by selected miRs, these results further emphasize the importance of studying the role of miRs in the AD pathology with due attention to the sex-specific differences.

## Electronic supplementary material

Supplementary Fig. 1 (PNG 427 kb)

High resolution image (TIF 817 kb)

Supplementary Fig. 2 (PNG 103 kb)

High resolution image (TIF 464 kb)

Supplementary Fig. 3 (PNG 261 kb)

High resolution image (TIF 605 kb)

ESM 1 (DOCX 16 kb)
