# Supplementary figures and images for: Selected microRNAs Increase Synaptic Resilience to the Damaging Binding of the Alzheimer’s Disease Amyloid Beta Oligomers
Source: Mol Neurobiol. 2020 Jan 29;57(5):2232–43. doi: 10.1007/s12035-020-01868-8 (PMC7170988; doi:10.1007/s12035-020-01868-8)

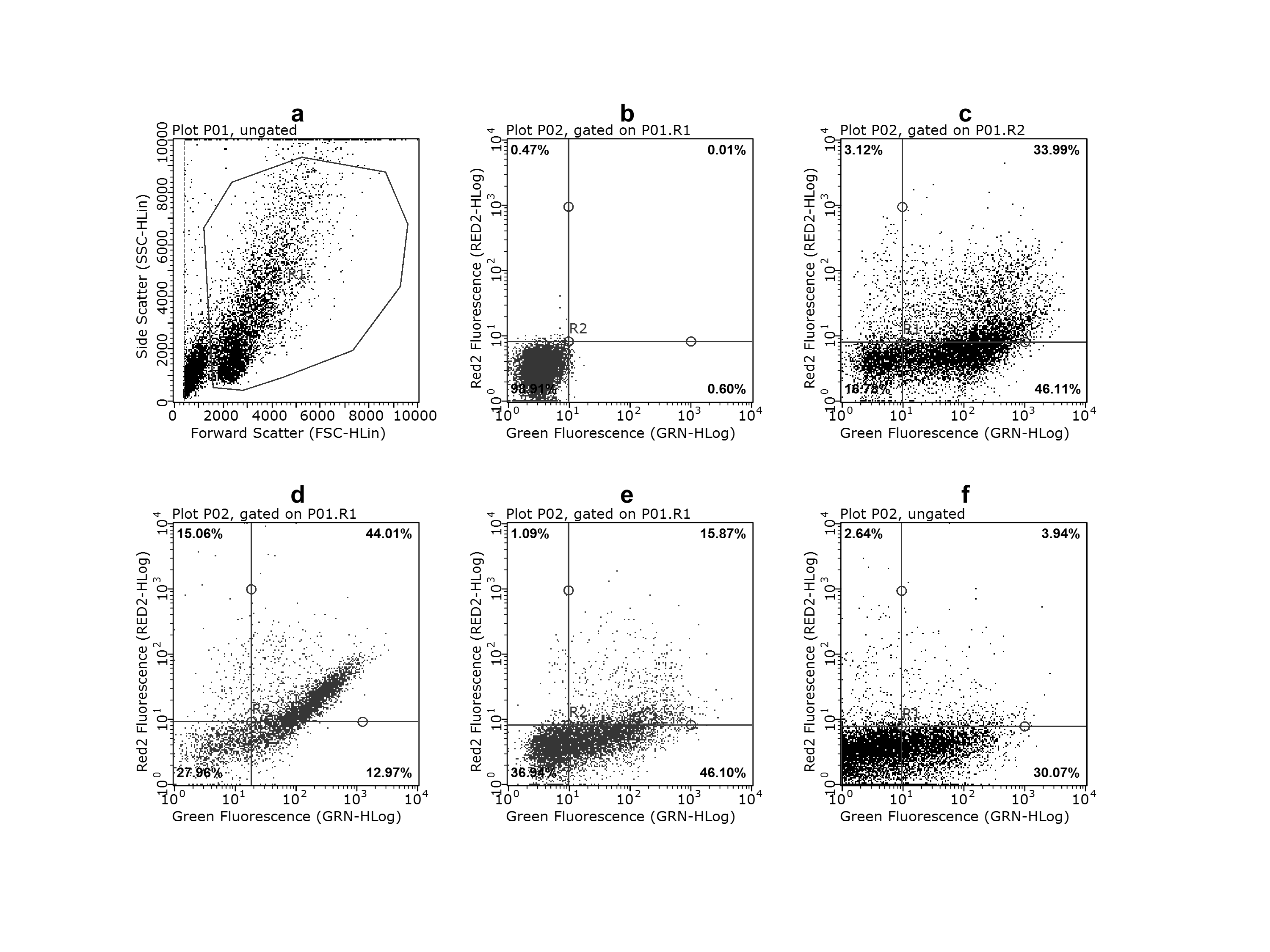

Supplement: Supplementary file 1 — (PNG 427 kb) [file 12035_2020_1868_Fig7_ESM.png]

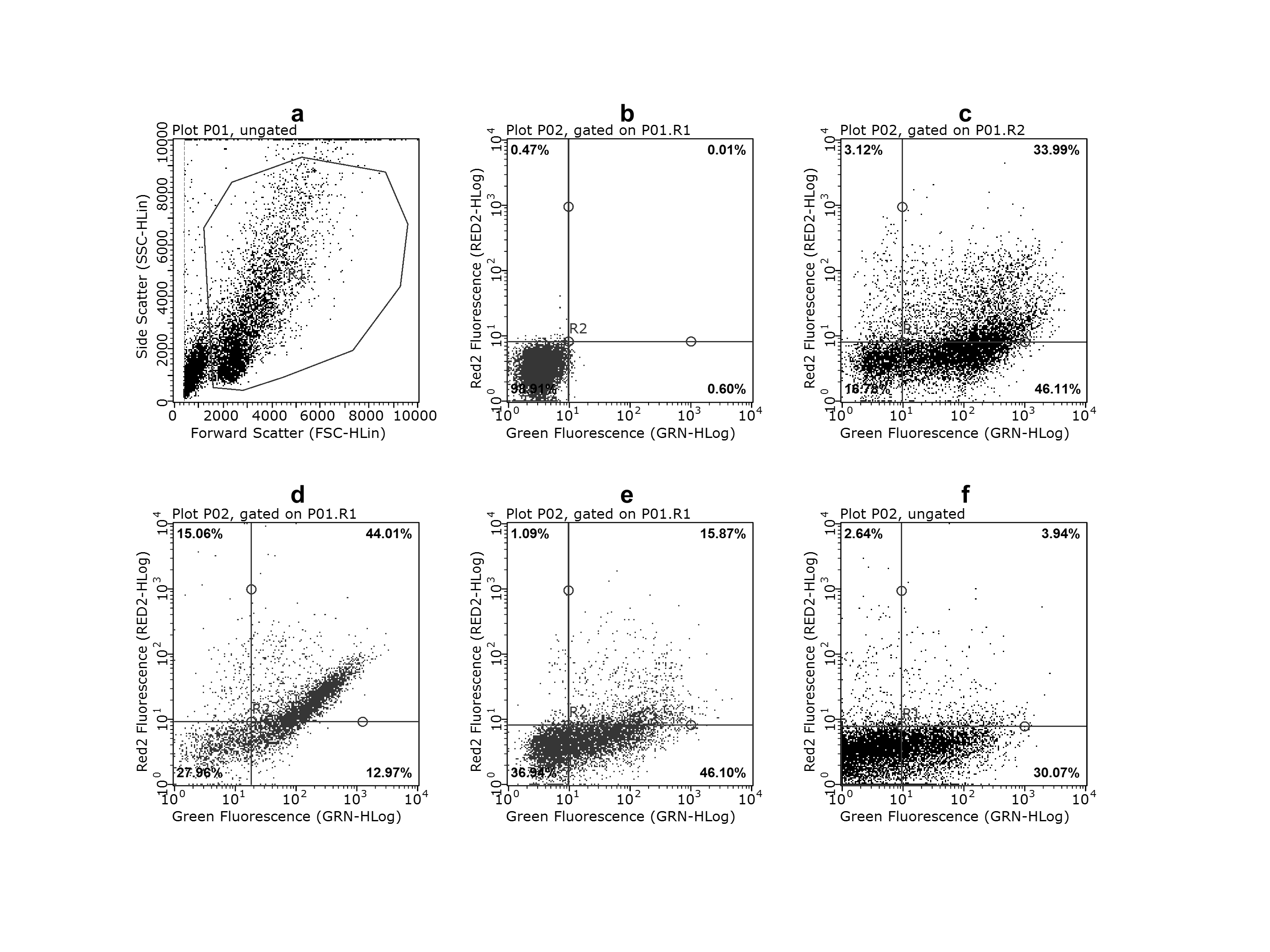

Supplement: Supplementary file 2 — High resolution image (TIF 817 kb) [file 12035_2020_1868_MOESM1_ESM.tif]

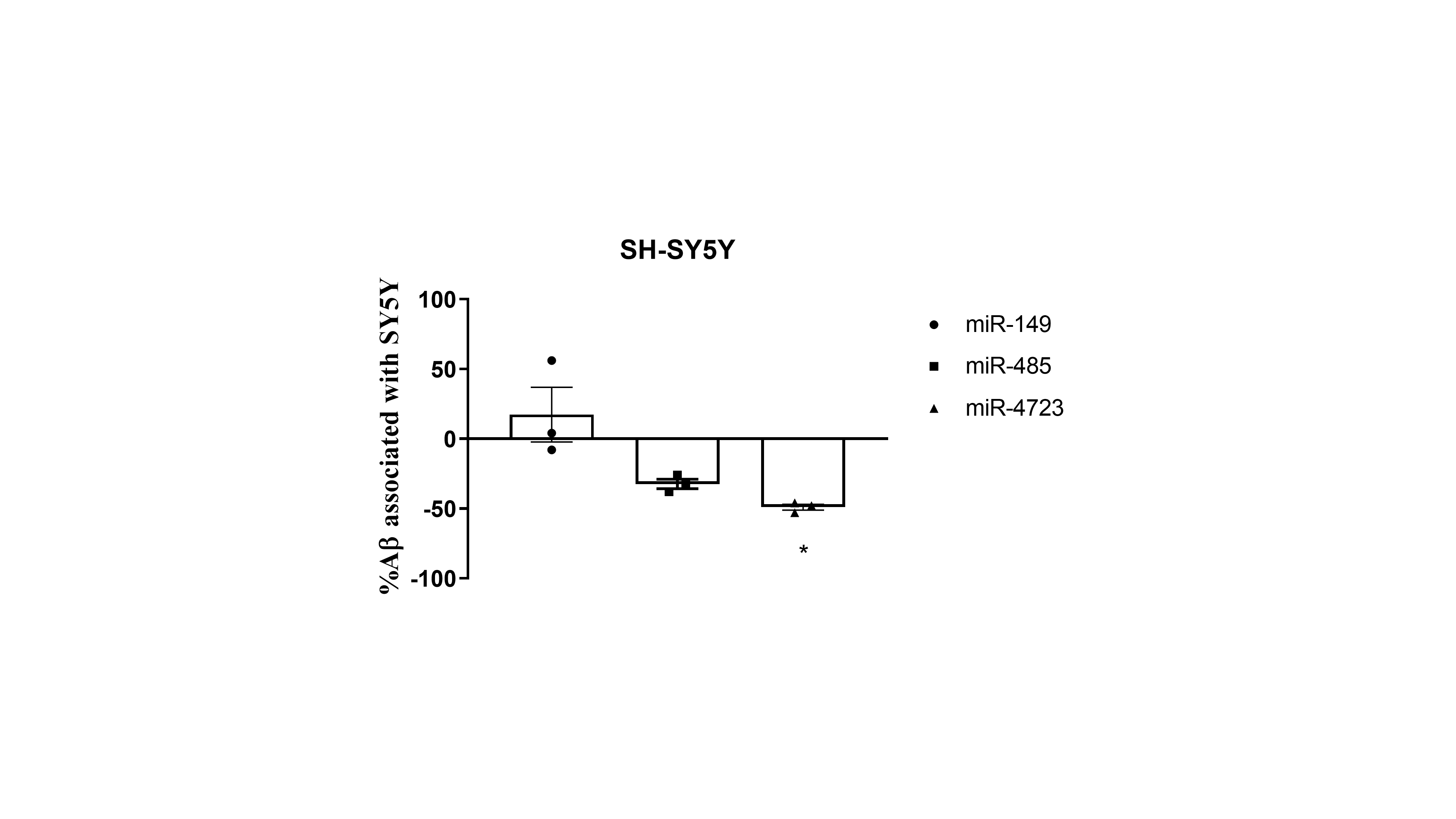

Supplement: Supplementary file 3 — (PNG 103 kb) [file 12035_2020_1868_Fig8_ESM.png]

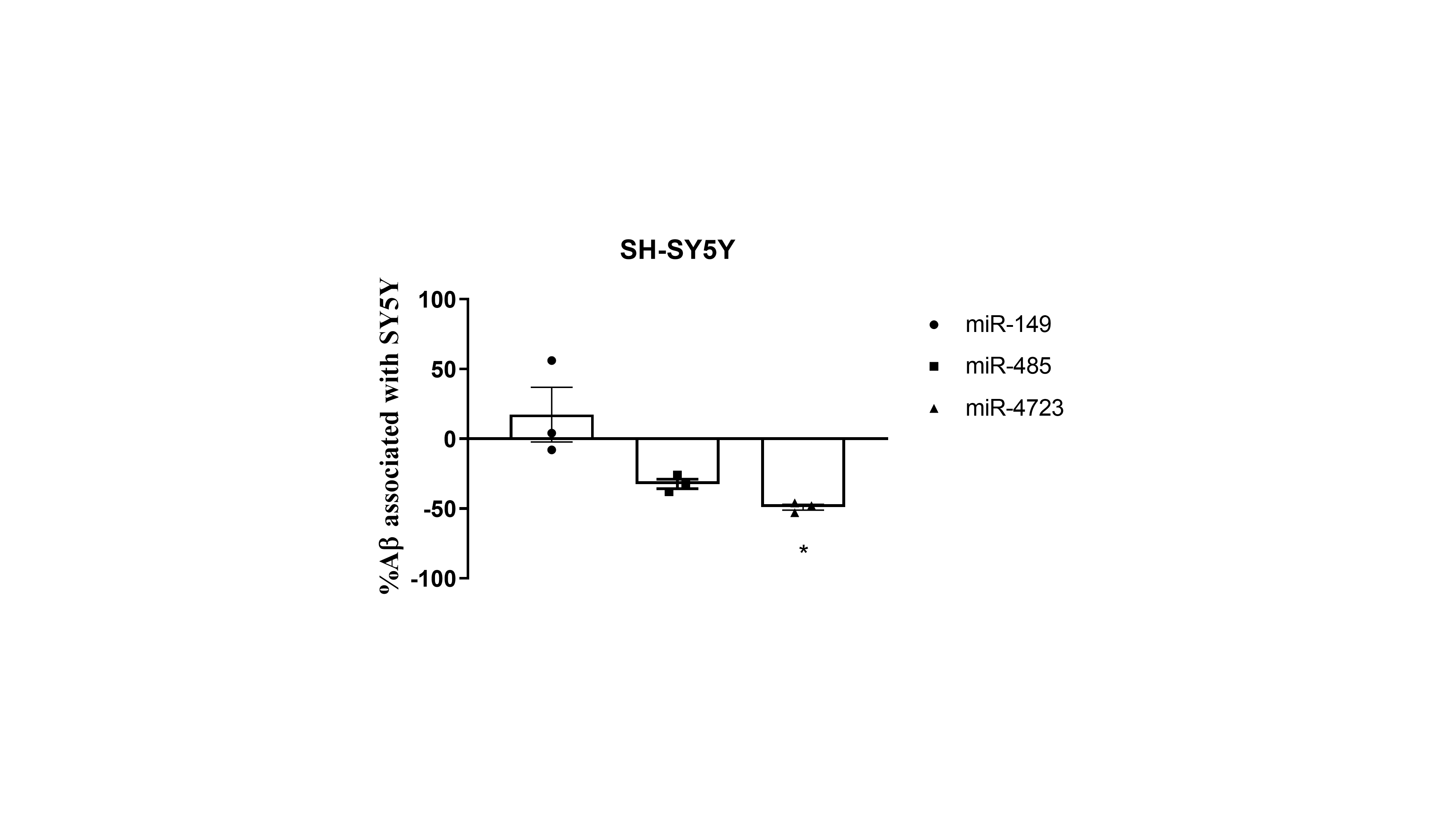

Supplement: Supplementary file 4 — High resolution image (TIF 464 kb) [file 12035_2020_1868_MOESM2_ESM.tif]

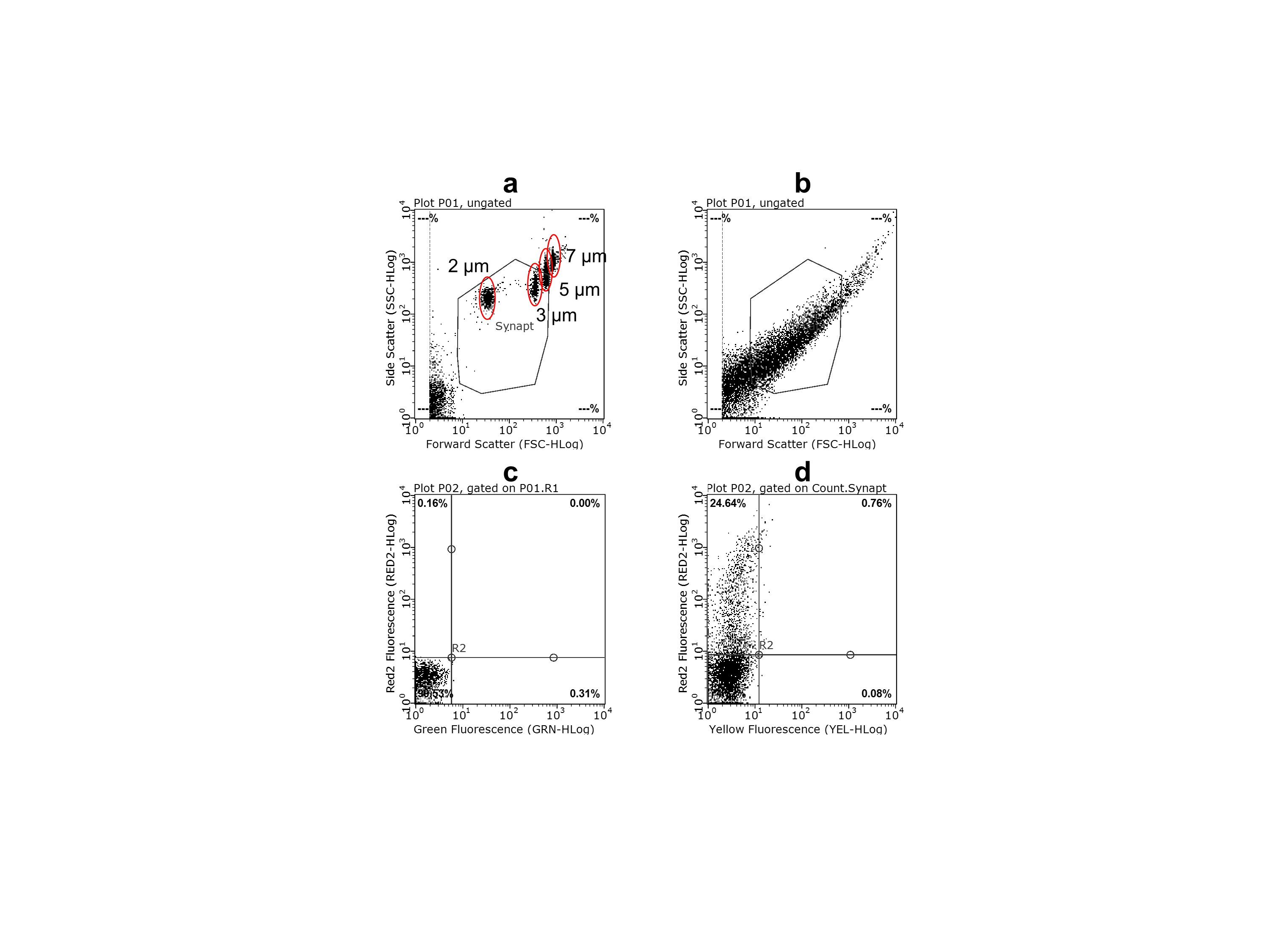

Supplement: Supplementary file 5 — (PNG 261 kb) [file 12035_2020_1868_Fig9_ESM.png]

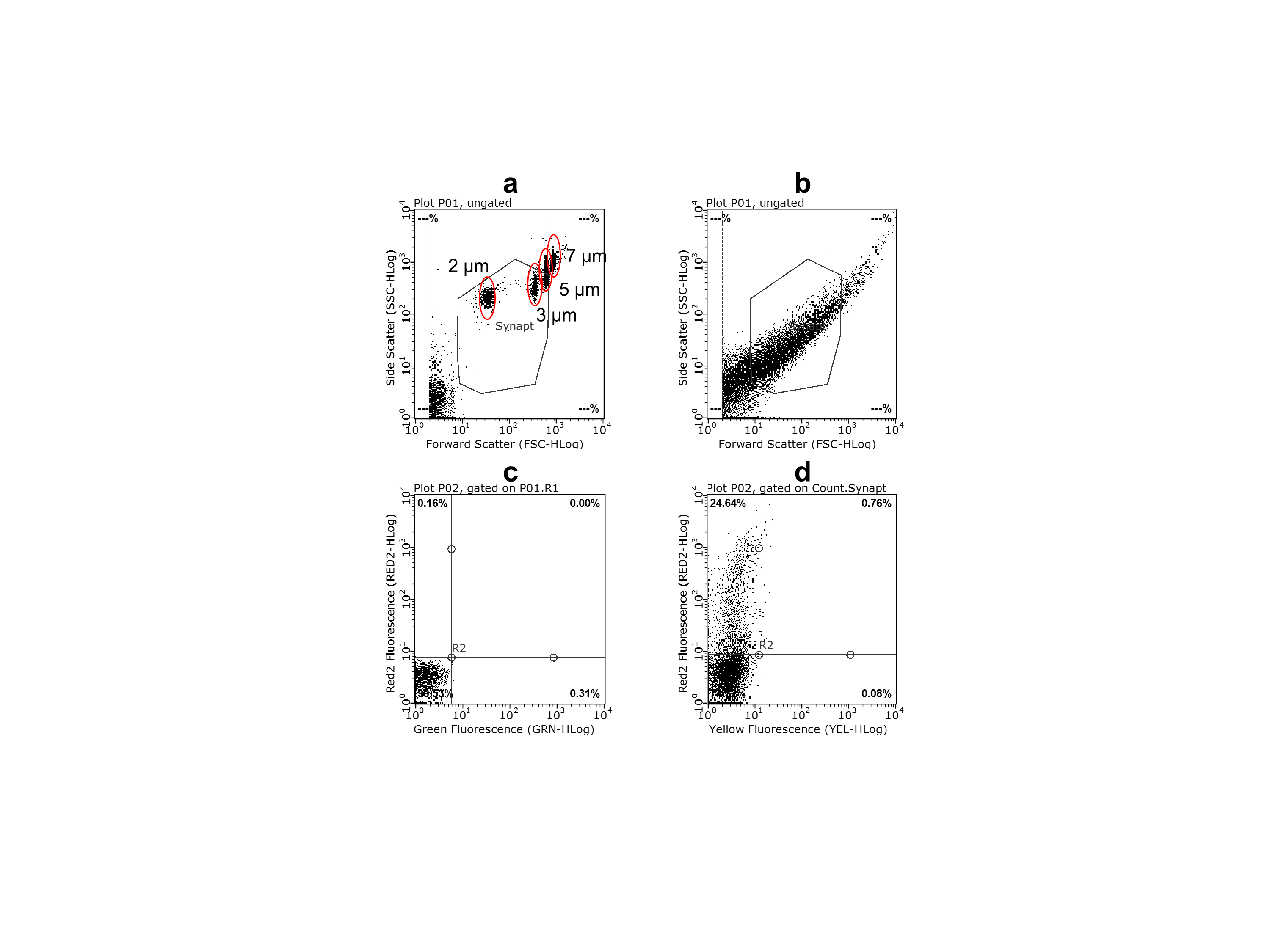

Supplement: Supplementary file 6 — High resolution image (TIF 605 kb) [file 12035_2020_1868_MOESM3_ESM.tif]
