# Supplementary material for: Selected microRNAs Increase Synaptic Resilience to the Damaging Binding of the Alzheimer’s Disease Amyloid Beta Oligomers
Source: Mol Neurobiol. 2020 Jan 29;57(5):2232–43. doi: 10.1007/s12035-020-01868-8 (PMC7170988; doi:10.1007/s12035-020-01868-8)
Supplement: Supplementary file 7 — (DOCX 16 kb) [file 12035_2020_1868_MOESM4_ESM.docx]

Supp. Table 1

Number of mRNAs by category from the PANTHER molecular function analysis of the hippocampal transcriptome in females. Number provided in this table represent the number of mRNAs falling into each category

|  | Females | | | Males | | |
| --- | --- | --- | --- | --- | --- | --- |
| Biological process | miR-149 | miR-485 | miR-4723 | miR-149 | miR-485 | miR-4723 |
| binding | 241 | 302 | 51 | 4 | 101 | 27 |
| catalytic activity | 190 | 204 | 52 | 8 | 90 | 24 |
| molecular function regulator | 50 | 60 | 14 | 1 | 25 | 9 |
| molecular transducer activity | 51 | 90 | 12 | 2 | 30 | 9 |
| structural molecule activity | 25 | 22 | 3 | 1 | 14 | 4 |
| transcription regulator activity | 1 | 53 | 13 | 0 | 17 | 2 |
| transporter activity | 57 | 62 | 18 | 16 | 24 | 12 |

Supp. Table 2

Synaptic genes measured in mice treated with miR-149, miR-485 and miR-4723. mRNA descriptions were obtained from the NCBI Gene database

| mRNA | Brief description |
| --- | --- |
| App | amyloid precursor protein |
| Bace1 | beta-secretase 1 |
| Camk2a | calcium/calmodulin-dependent protein kinase II alpha |
| Creb1 | cAMP responsive element binding protein 1 |
| Dnm1 | dynamin 1 |
| Mapt | microtubule-associated protein tau |
| Ppp3ca | protein phosphatase 3, catalytic subunit, alpha isoform |
| Psd95 | postsynaptic density protein 95 |
| Snap25 | synaptosomal-associated protein 25 |
| Snca | synuclein, alpha |
| Syn1 | synapsin I |
| Vamp2 | vesicle-associated membrane protein 2 |
